# The Impacts of Cellular Senescence in Elderly Pneumonia and in Age-Related Lung Diseases That Increase the Risk of Respiratory Infections

**DOI:** 10.3390/ijms18030503

**Published:** 2017-02-25

**Authors:** Shigehisa Yanagi, Hironobu Tsubouchi, Ayako Miura, Ayako Matsuo, Nobuhiro Matsumoto, Masamitsu Nakazato

**Affiliations:** Division of Neurology, Respirology, Endocrinology and Metabolism, Department of Internal Medicine, Faculty of Medicine, University of Miyazaki, Kihara 5200, Kiyotake, Miyazaki 889-1692, Japan; hironobu_tsubouchi@med.miyazaki-u.ac.jp (H.T.); ayako_miura@med.miyazaki-u.ac.jp (A.Mi.); ayako_matsuo@med.miyazaki-u.ac.jp (A.Ma.); nobuhiro@med.miyazaki-u.ac.jp (N.M.); nakazato@med.miyazaki-u.ac.jp (M.N.)

**Keywords:** elderly pneumonia, aging, cellular senescence, senescence-associated secretory phenotype, antimicrobial defense system, chronic obstructive pulmonary disease, idiopathic pulmonary fibrosis

## Abstract

Pneumonia generates considerable negative impacts on the elderly. Despite the widespread uses of vaccines and appropriate antibiotics, the morbidity and mortality of elderly pneumonia are significantly higher compared to the counterparts of young populations. The definitive mechanisms of high vulnerability in the elderly against pathogen threats are unclear. Age-associated, chronic low-grade inflammation augments the susceptibility and severity of pneumonia in the elderly. Cellular senescence, one of the hallmarks of aging, has its own characteristics, cell growth arrest and senescence-associated secretory phenotype (SASP). These properties are beneficial if the sequence of senescence–clearance–regeneration is transient in manner. However, persisting senescent cell accumulation and excessive SASP might induce sustained low-grade inflammation and disruption of normal tissue microenvironments in aged tissue. Emerging evidence indicates that cellular senescence is a key component in the pathogenesis of chronic obstructive pulmonary disease (COPD) and idiopathic pulmonary fibrosis (IPF), which are known to be age-related and increase the risk of pneumonia. In addition to their structural collapses, COPD and IPF might increase the vulnerability to pathogen insults through SASP. Here, we discuss the current advances in understanding of the impacts of cellular senescence in elderly pneumonia and in these chronic lung disorders that heighten the risk of respiratory infections.

## 1. Introduction

Pneumonia causes significant mortality and morbidity in elderly patients, defined as those aged over 65 years, compared to younger populations [1,2,3,4,5]. The annual incidence of pneumonia in the elderly populations is 4 times that of younger populations [6]. In addition, the rates of hospitalization for pneumonia increase in elderly patients with each passing year [2], and with an expected 20% of the world’s population reaching elderly status by 2050, the burden of community-acquired pneumonia will be even more significant in the coming years [7]. Furthermore, hospitalization for pneumonia has a considerable effect on economic burden, particularly for the elderly population [8]. Despite these continuing concerns and the widespread use of vaccines and appropriate antibiotics, the prognosis of elderly pneumonia remains dismal, and specific strategies to clear the vulnerability to pathogen threats in older individuals have not been proven.

Aging, defined as a time-dependent functional decline that affects most living organisms [9], causes progressive loss of physiological integrity, impaired organ function, and subsequent increased vulnerability to death [9]. In the respiratory system, aging might render individuals more susceptible to infection by undergoing various physiological changes, including dilatation of airspaces, increased air trapping, decreased chest wall compliance, reduced respiratory strength, decline in mucociliary clearance, and diminishment of cough reflex [3,10,11,12,13]. In addition, aging weakens the immune system in conjunction with the presence of comorbid diseases (e.g., diabetes mellitus, chronic heart disease, malignant tumors, and use of immunosuppressive drugs) [2,3,5,14,15,16]. However, the definitive mechanisms underlying the high morbidity and mortality of pneumonia in elderly populations are not fully understood.

Several lines of evidence indicate that age-associated, nonmicrobial, and chronic low-grade inflammation enhances the susceptibility of pneumonia in the elderly populations. A previous study reported that elevated tumor necrosis factor (TNF)-α and interleukin (IL)-6 levels positively correlated with the incidence of pneumonia in healthy elderly individuals [17]. Other studies demonstrated that aged mice had increased lung inflammation and were found to be highly susceptible to pneumococcal pneumonia [18,19]. Furthermore, young mice infused with age-relevant physiological levels of TNF-α for 5 days by using a subcutaneously implanted osmotic pump had 100-fold more *Streptococcus pneumoniae* in their lungs and 10-fold more bacteria in their blood than control mice one day after intranasal infection with *S. pneumoniae* [19].

Cellular senescence, one of the hallmarks of aging [20,21,22,23], carries out its primary duty as a trigger of tissue repair [24], regeneration [25], and remodeling during normal embryonic development [26,27,28] and upon tissue damage [29]. To eliminate damaged cells, senescent cells arrest their own proliferation, create an inflammatory microenvironment, recruit phagocytic immune cells for elimination of senescent cells through senescence-associated secretory phenotype (SASP) [30], and promote tissue renewal [24,29]. These processes are beneficial for organisms in young tissue where the sequence of senescence–clearance–regeneration is transient in manner [29]. However, this beneficial processes can be corrupted in a pathological context and aged tissues, where senescent cells persistently accumulate [29]. The combination of senescent cell accumulation and excessive SASP results in persistent low-grade inflammation in aging tissue [29,31], which elevates the susceptibility to pathogen threats. Furthermore, accumulation of senescent cells causes disruption of normal tissue microenvironments and aberrant tissue remodeling through extracellular matrix (ECM) degeneration and tissue fibrosis [29,31,32].

In the respiratory system, emerging evidence indicates that cellular senescence is a key component in the pathogenesis of chronic obstructive pulmonary disease (COPD) and idiopathic pulmonary fibrosis (IPF), which are known to be age-related diseases and increase the vulnerability to pneumonia [33,34]. Both of the diseases bear the feature of chronic low-grade inflammation with upregulations of various growth factors and chemokines [35,36]. Thus, it is speculated that COPD and IPF might enhance the vulnerability to pathogens not only by their structural collapse of lung parenchyma, which makes it easier for pathogens to invade, but also by inducing chronic low-grade inflammation due to SASP. Since both of the lung disorders predominantly affect the elderly [34,37] and have a lot of involvement in the susceptibility to the pathogens, we contemplate that it is also important to focus on the involvement of cellular senescence in the pathogenesis COPD and IPF for getting to the core of the pathomechanism of elderly pneumonia. In this review, we highlight the impacts of cellular senescence on the pathogenesis of pneumonia and in age-related lung diseases that increase the risk of respiratory infections. We first describe the machinery and impact of the cellular senescence in aged tissue. We next discuss the impacts of aging in respiratory tract antimicrobial defense system. Finally, we discuss the role of cellular senescence in the pathogenesis of COPD and IPF.

## 2. Cellular Senescence, SASP, and Aging

### 2.1. Cellular Senescence

The first formal description of cell senescence was made more than five decades ago, when Leonard et al. showed that normal human fibroblasts had a finite proliferative capacity in culture, and speculated that this cell property could be linked to aging [20]. Cellular senescence can be defined as an irreversible arrest of cell proliferation coupled to induction of the multicomponent secretory phenotype, SASP. Cellular senescence primarily acts as an irreplaceable defense against cancer progression by preventing unrestricted cell growth of damaged cells. Cellular senescence occurs when cells encounter various kinds of stressors and stimuli including DNA damage and mutations, telomere shortening, oxidative stress, oncogene activation, tumor suppressor loss, and epigenomic stress [29,31]. Senescent cells reorganize chromatin, resulting in heterochromatin formation, extensive gene expression changes, and changes in cell and organelle shape [29,38]. Senescent cells are relatively resistant to apoptosis, and can be efficiently cleared thorough macrophage-mediated phagocytosis [39,40]. Emerging evidence demonstrates that the role of cellular senescence extends beyond tumor suppression to biological processes including embryonic development [26,27,28], wound healing [25], tissue repair [24], and aging [22,23].

Senescent cells are characterized by several properties, markers, and morphological changes. These characteristics include (1) the absence of proliferative markers (e.g., Ki67, 5-bromodexyuridine incorporation); (2) senescence-associated β-galactosidase (SAβGAL) activity; (3) expression of tumor suppressors and cell cycle inhibitors (e.g., p16, ADP-ribosylation factor (ARF), p53, p21, p15, p27, and hypophosphorylated Rb); (4) senescence-associated heterochromatic foci; and (5) enlarged or flat cell morphology [29]. In addition, senescent cells secrete a number of extracellular factors, including transforming growth factor-β1 (TGF-β1), insulin-like growth factor 1 binding protein (IGFBP), and various inflammatory cytokines and chemokines [29,31].

Among these properties, the most widely employed for cellular senescence is histochemical detection of SAβGAL activity at pH 6.0 [41]. This activity is thought to reflect an increase in lysosomal enzyme mass in senescent cells [42]. The increase of lysosomal content is not simply due to the results of the general increase in cytoplasmic constituents, as demonstrated by the fact that β-galactosidase protein content increased even when measured relative to actin [42], but reflect the malfunctional change of senescent cells. It has been suggested that the increase of cellular lysosomal content in aging cells is the consequence of the accumulation of nondegradable intracellular macromolecule and organelles in autophagic vacuoles [42]. Accumulation of undigested material (lipofuscin) inside autolysosomes disturbs their ability to fuse with autophagosomes and to degrade their cargoes (i.e., damaged proteins and organelles) [43]. Thus, in aging tissue, the increase of lysosomal content is thought to be a result of dysfunctional autophagy, rather than that of increased autophagy. Since SAβGAL activity is not required for senescence [44], the increase of SAβGAL activity in senescent cells is thought to be an outcome rather than a cause of senescence.

Autophagy, a major lysosomal degradation pathway, plays an important role in maintenance of cellular homeostasis by removing misfolded or aggregated proteins and clearing damaged organelles, including mitochondria (i.e., mitophagy) [43]. Autophagy has the capacity for both selective or nonselective engulfment of bulk cytoplasmic proteins or organelles, and can limit production of reactive oxygen species [43,45]. Failure to degrade unfolded proteins can lead to their accumulation and aggregation, resulting in proteotoxic effects [9]. Impairment in the regulation and conduct of macroautophagy, in which the cargoes are sequestrated within a unique double-membrane cytosolic vesicle that later fused with lysosomes [43], contribute to functional decline during aging [46]. The activity of the autophagy–lysosomal system declines with aging [46,47], and defective autophagic function has been reported in almost all tissues of aging organisms [43,45]. In accord with these observations, induction of macroautophagy increases longevity in mice [48], and inhibition of autophagy induces functional deterioration and age-related pathologies [49,50,51]. Thus, autophagy primarily functions as cytoprotective process, and insufficient autophagy contributes to, at least in part, aging and aging-associated phenotypes.

Cellular senescence and autophagy are distinct (but closely related) cell properties, and both are important for homeostatic stress responses. The precise mechanisms by which autophagy positively or negatively regulates cellular senescence is still under debate. In consideration of the relation between autophagy and cellular senescence, it is thought to be important to consider the levels of autophagy (e.g., basal level or induced level), the mode of autophagy (e.g., general autophagy or selective autophagy) [52], and the type of cellular senescence. Autophagy is induced when needed, but apart from that, it is maintained at a basal level [43]. The basal level of autophagy functions as a protective pathway for normal cell homeostasis. Autophagy inhibition promotes cellular senescence in normal proliferating cells [53,54]. On the other hand, stress of oncogene activation triggers autophagy induction and facilitates the process of senescence [55]. Dou et al. recently reported that oncogene hyperactivation induces selective autophagy which targets lamin B1, a component of nuclear lamina [56]. This autophagic degradation of lamin B1 results in oncogene-induced senescence and subsequent tumor suppression [56]. A recent study also demonstrated that selective autophagy, which specifically degrades GATA4, a key regulator of the SASP and cellular senescence during aging, prevents cellular senescence [57]. These observations regarding selective autophagy may provide an explanation for the difficult question of how autophagy positively or negatively regulates cellular senescence. Nonetheless, because the cross-talk between cellular senescence and autophagy is quite complex, it needs more investigations to understand the accurate interrelationship between these two cell processes.

At present, there are no markers or properties that are completely specific or universal for all senescence types. Nevertheless, it is important to note that most of the above senescence markers have been validated in vivo, both in association with premalignant tumors and in association with developmental, physiological, and pathological processes [29].

Multiple stresses activate the cell senescence program. These stresses are signaled through various pathways, but finally activate the p53–p21 pathway or p16 or both [29,31]. Both p53–p21 pathway and p16 converge on the inhibition of cyclin-dependent kinase (CDK)–cyclin components, prevents the inactivation of Rb, and results in cell-cycle arrest. The relative contribution of the p53–p21 pathway and p16 to cell-cycle arrest may vary depending on the cell type as well as the type of senescence triggers and functional nature (e.g., damage-induced senescence, oncogene-induced senescence, developmentally programed senescence).

Senescence growth arrest exhibits beneficial effects on prevention of precancerous cells [40,58,59] and attenuation of skin and liver fibrosis [24,25]. On the other hand, senescence growth arrest also occurs on stem or progenitor cells [29,31], in which case they lose the ability to proliferate for tissue regeneration and repair. For example, senescence of muscle stem cells is thought to be an underlying cause of aging-associated sarcopenia and loss of muscle regenerative potential [60,61].

### 2.2. SASP

On an equal footing with cell-cycle arrest, SASP is an important feature of senescent cells. Interestingly, normal cells that senesce owing simply to the ectopic induction of p21 or p16 do not express an SASP, despite undergoing a senescence growth arrest [62]. In contrast, cells that senesce owing to DNA damage, mitogenic signals, oxidative stress, and other senescence-inducing stress develop an SASP [63]. These findings suggest that the role of SASP may be (1) a communication tool of damaged cells to neighboring cells about the information of their compromised status for preparation of the tissue repair and (2) stimulation of the immune system to clear the damaged cells from the tissue [63].

The SASP activation is positively regulated by DNA damage response, nuclear factor-κB (NF-κB), and CCAAT/enhancer-binding protein β [29,63], and is negatively regulated by p53 [63]. SASP components include many proinflammatory cytokines (e.g., IL-6 and IL-8), chemokines (e.g., monocyte chemoattractant proteins and macrophage inflammatory proteins), growth factors (e.g., TGF-β1, IGFBP, vascular endothelial growth factors, and granulocyte-macrophage colony stimulating factor), and proteases (e.g., matrix metalloproteinase (MMP)-1, -2, -3). SASP factors vary in distinct cell types and under different senescence-inducing stimuli [31]. On the other hand, proinflammatory cytokines and chemokines are among the SASP components that are highly conserved features, cutting across many different cell types and senescence-inducing stimuli [31,63]. Thus, SASP factors might intimately contribute to signaling for migration of phagocytes that play important roles in the clearance of senescent cells and the regeneration of damaged tissue [29,38]. SASP components also have the potential to modulate the tissue microenvironment through various biological processes including cell proliferation, cell migration, inflammation, fibrosis, degradation of ECM, neovascularization, and epithelial–mesenchymal transition in paracrine and autocrine manners [31]. TGF-β1, a notable component of SASP, leads senescence in neighboring cells in a paracrine manner via upregulation of the cell cycle inhibitors p21, p27, and p15 through the Smad signaling pathway [29]. Similarly, IL-6 and IL-8 secreted by senescent cells can trigger paracrine senescence in bystander cells [64,65]. Furthermore, the stimulation of the IL6R/NF-κB pathway cooperates with TGF-β1/Smad to induce bystander senescence [66]. Thus, SASP has powerful reinforcement activities in a cell-autonomous and bystander activation manner to develop the inflammatory microenvironment for the elimination of senescent cells.

As with the senescence growth arrest, SASP can be beneficial or deleterious, with the difference depending on whether SASP is transiently or chronically present overtime. A localized, transient SASP is important for recruitment of immune cells, clearance of damaged cells, and subsequent regeneration/remodeling of tissue, at least in young tissue and in developmental transitory embryonic structures [29,31]. However, after persistent damage, pathological status, or in aged tissue, clearance of senescent cell and regeneration may be compromised. In these situations, senescent cells accumulate and subsequent persistent SASP causes chronic low-grade inflammation and tissue dysfunction [29,31]. In addition, whereas senescent cells in tumors can recruit immune cells through the SASP and induce tumor clearance [40], high burden of senescent cells and persistent SASP induce immune suppression and tumor promotion [67].

### 2.3. The Role of Cellular Senescence in Aging and Aging-Related Diseases

The accumulations of senescent cells with chronological aging tissue and in progeroid syndromes have been observed in multiple mammalian organs, including the lungs [41,68,69,70,71,72,73]. Accumulation of senescent cells might be induced by several proposed mechanisms including (1) increased generation of senescent cells partly by aggressive SASP; (2) decreased clearance of senescent cells due to impaired immune system; and (3) high resistance of senescent cells against immune clearance [29,31,74].

The elegant study by Baker et al. revealed that eliminating senescent cells can delay age-related dysfunction in a progeroid rodent model [22]. This is the first evidence that senescent cells are direct drivers of multiple age-related pathologies. Recent studies have developed several transgenic mouse models that enable investigations about the emergence of senescence cells in vivo and their roles in driving aging phenotypes and age-related diseases. These models use the promoters of p21 or p16 to drive the expression of reporter and/or killer genes by senescent cells [23,75,76,77,78]. The senescence-reporter models demonstrated that senescent cells increase in number during chronologic aging [23,77,78]. Baker et al. reported on the direct contribution of cellular senescence in aging phenotypes by using the model of selective elimination of senescent cells in vivo [23]. The clearance of senescent cells results in extended life span, delayed tumorigenesis, and mitigated age-related deterioration of several organs, including kidney, heart, and fat [23]. These studies shed a ray of light on the elucidation of the direct contribution of senescent cells on aging and aging-related diseases.

To date, there are five possible scenarios by which senescent cells promote age-related tissue dysfunction. First, cellular senescence can deplete the stem or progenitor cells from the tissue [29,31,38]. This condition decreases tissue repair and regeneration capacity. Second, the senescence impairs the efficacy of reprogramming of somatic cells into induced pluripotent stem cells [79,80]. Third, senescence could disrupt the local stem cell niche non-autonomously through the SASP and could negatively impact on stem cell function [31]. Fourth, inappropriate presence of SASP components could functionally and structurally perturb normal tissue microenvironment. Persistent induction of SASP components causes degradation of ECM, induction of aberrant cell differentiation, tissue fibrosis, and stimulation of low-grade chronic inflammation [31,38]. Finally, SASP components (e.g., TGF-β1) could cause paracrine senescence in healthy bystander cells, increase the population of senescent cells, and reinforce age-related tissue deterioration.

## 3. The Impacts of Aging in Respiratory Tract Antimicrobial Defense System

Aging has a wide spectrum of defective impacts on both innate and adaptive immunity, including the functions of alveolar macrophages, dendritic cells, and neutrophils, and the mechanisms involved have been extensively reviewed [81,82,83]. In regard to the antimicrobial peptide production, the levels of cathelicidin and β-defensin-2 in healthy elderly were comparable with those found in healthy young individuals [84]. In contrast, aging causes slowing in ciliary beat frequency in mice [85] that may lead to diminished mucociliary clearance for elimination of pathogens from the airway. Aging also causes impaired alveolar barrier integrity after lung injury. In comparison with young mice, old mice had increased response of acute lung injury in association with decreased expressions of tight junction protein after lipopolysaccharide insult [86]. Yin et al. demonstrated that aged mice had increased susceptibility to influenza viral pneumonia with exacerbated damage and delayed repair of alveolar epithelial cells (AECs) [87]. Previous studies reported that signaling of Toll-like receptors (TLRs), pattern recognition receptors for detection and initiation of innate immune response, were impaired in aging [88,89,90]. Defective TLR signaling in the elderly might lead to impairment of rapid recognition of pathogens, and might contribute to increased baseline levels of inflammation. Elderly individuals have baseline low-grade chronic inflammation, known as “inflamm-aging”, in the absence of an infectious insult [81,82]. Blunted immune response, referred as immunosenescence, might contribute to the development of inflamm-aging in elderly. In addition, the accumulations of senescent cells in aged tissues might also contribute to the evolution of low-grade chronic inflammation. Interestingly, Shivshankar et al. reported that aged mice had increased bacterial ligand expression, and enhanced susceptibility to pneumococcal pneumonia with elevated levels of senescence markers in the lung [18]. These findings suggest that cellular senescence might contribute directly to the susceptibility of elderly to bacterial infection.

## 4. The Impacts of Cellular Senescence in Age-Related Lung Diseases

### 4.1. The Role of Cellular Senescence in COPD

COPD is characterized by persistent airflow limitation that is usually progressive and associated with an enhanced chronic inflammatory response in the lungs [37]. COPD predominantly affects the elderly, with the peak prevalence at approximately 65 years old [37]. COPD patients have a chronic inflammatory response in the lungs, and this response might induce emphysema and obstruction of the small airways [37]. Patients with COPD have an increased risk of community-acquired pneumonia [33].

For a long period of time, inhalation of toxic particles and gases, primarily cigarette smoke, is thought to play a central role in the development of chronic inflammatory response in COPD patients. However, this scenario cannot fully explain the development of chronic inflammation in the lungs, because persistent airway inflammation and progression of disease are observed in patients with COPD who have already ceased smoking [91]. This observation, in conjunction with the high prevalence of COPD in elderly, suggests the involvement of cellular senescence in the pathogenesis of COPD. Several studies indicated the existence of shortened telomeres in various type of cells in patients with COPD, including type II AECs, fibroblasts, endothelial cells, and peripheral blood lymphocytes [92,93,94,95]. Previous studies also showed cigarette smoke induction of cellular senescence in AECs and fibroblasts with the expressions of SAβGAL in vitro and in vivo [96,97]. In addition, multiple SASP components—including IL-6, IL-8, and MMPs—are upregulated through the activation of NF-κB, and are closely linked to the pathogenesis of COPD [35].

The level of anti-aging sirtuin 1 (SIRT1), a NAD+-dependent protein/histone deacetylase, was low in alveolar macrophage and epithelial cells in the lungs of patients with COPD [98]. SIRT1 activation by genetic overexpression and a pharmacological SIRT1 activator mitigated cigarette smoking-induced and elastase-induced emphysema with the reduction in number of SAβGAL-positive epithelial cells in mice [99]. In accordance with these findings, deletion of SIRT1 resulted in aggravated emphysematous change with the increased number of SAβGAL-positive epithelial cells after cigarette smoke challenge in mice [99]. These findings indicated that cellular senescence is a key player in the pathogenesis of COPD, and activation of SIRT1 might be an attractive therapeutic strategy against COPD.

Recently, Richmond et al. reported that polymeric immunoglobulin receptor-deficient mice, which lack secretory immunoglobulin A, spontaneously developed fragmentation of alveolar wall and small airway fibrosis with the activation of NF-κB associated with chronological aging [100]. These mice showed an altered lung microbiome, increased bacterial penetration into bronchial epithelium, enhanced macrophage accumulation, and increased MMP-12 levels [100]. Interestingly, re-derivation of these mice in germ-free conditions protected them from chronic lung inflammation and emphysema. Since downregulation of polymeric immunoglobulin receptor correlates with airway inflammation in patients with COPD [101], alteration of the microbiome might contribute to the progression of COPD, and cellular senescence might collaborate with an altered airway microbiome in development of the vicious circle of low-grade chronic lung inflammation in COPD.

### 4.2. The Role of Cellular Senescence in IPF

IPF is defined as specific form of a chronic, progressive, age-associated, fatal, irreversible, and fibrosing interstitial pneumonia of unknown causes, occurring primarily in older adults [34]. The incidence of disease increases with older age, with presentation typically occurring in the sixth and seventh decades [102,103,104]. The most significant environmental risk factor is cigarette smoking [34,102]. IPF patients are considered to have increased risk of respiratory infection due to receiving immunosuppressive drugs and therapy and repeated hospitalization [34]. From the perspective of histopathologic characteristics of IPF, two essential properties of senescent cells, cell-cycle arrest and SASP, are thought to be intimately involved in the pathogenesis of IPF. Reconstitution of AEC-barrier integrity might fail due to cell-cycle arrest of AECs, and that might result in proliferation and activation of fibroblast, surplus collagen deposition, and fibrotic scarring. In addition, accumulation of fibroblasts adjacent to large or flattened AECs in fibroblastic foci suggest the disruption of normal epithelial–mesenchymal interaction due to excessive SASP components originated from senescent cells. In fact, SAβGAL-positive senescent cells are frequently detected in both AECs [105,106,107] and fibroblasts [105] in fibroblastic foci of lungs affected by IPF. The expressions of CDK inhibitors (p16, p21, and p53) were elevated in type II AECs isolated from IPF patients [107]. In addition, short telomeres were also detected in AECs of lungs affected by IPF [108]. Furthermore, AECs of lungs affected by IPF are the primary source of various SASP components that act as chemotactic factors, mitogens, or ECM remodelers, including TGF-β1, MMPs, and IGFBP [36]. Among these, TGF-β1 plays a pivotal role in the pathogenesis of IPF through the induction of migration, proliferation, and activation of fibroblasts, and evocation of epithelial mesenchymal transition in AECs [36].

Recent transgenic mouse model studies demonstrated that cellular senescence in AECs has an intimate involvement in the pathogenesis of IPF. Type II AEC-specific deletion of type II telomere repeat binding factor (TRF)-1—a telomere shelterin protein—in mice resulted in short telomeres [109], increased expressions of p53 and p21 [110], accumulation of SAβGAL-positive senescent cells [109], increased TGF-β1 expression [109], and development of spontaneous lung fibrosis [109,110]. Importantly, short telomeres and accumulation of SAβGAL-positive senescent cells were detected only in older, but not in younger, TRF-1 deleted mice [109]. Alder et al. reported that type II AEC-specific TRF-2-deficient mice resulted in robust activation of DNA damage response, increased expressions of p53 and p21, decreased cell proliferation of type II AECs, increased spontaneous macrophage recruitment, and impaired lung repair after injury [111]. They also demonstrated that TRF-2 deletion in type II AECs limited self-renewal and differentiation of AECs in vitro [111]. These findings suggest that cellular senescence in AECs plays an important role in the pathogenesis of lung fibrosis through the cell growth arrest, SASP induction, and limitation of alveolar stem cell function.

In regard to fibroblasts in IPF, Yanai et al. demonstrated the accelerated replicative cellular senescence and large and irregular morphology with high frequent positivity for myofibroblast marker (α-smooth muscle actin) in lung fibroblasts isolated from patients with IPF [112]. Similarly, Im et al. reported the decreased autophagy activity in primary lung fibroblasts from patients with IPF [113].

Type II AECs in the lungs of patients with IPF showed defective mitophagy and dysfunctional mitochondria with low expressions of PTEN-induced putative kinase 1 (PINK1) that is thought to have an important role in the maintenance of mitochondrial homeostasis and selective degradation of damaged mitochondria by mitophagy [114]. PINK1-deficient mice showed dysfunctional mitochondria in type II AECs with increased vulnerability to lung fibrosis [114]. These findings suggest that impaired mitophagy in AECs promotes susceptibility to lung fibrosis. Conversely, alveolar macrophages from patients with IPF showed increased mitophagy and apoptosis resistance with increased expression of TGF-β1 [115]. Macrophage-specific Akt-deficient mice, which exhibit decreased mitochondrial reactive oxygen species, showed impaired mitophagy, had decreased TGF-β1 expressions, and were protected from lung fibrosis [115]. Taken together, mitophagy positively or negatively regulates the development of lung fibrosis dependent on the individual, cell-intrinsic property.

### 4.3. The Potential Scenarios of How Cellular Senescence Causes Distinct Age-Related Lung Pathologies

As described in above chapters, several evidences have indicated that cellular senescence plays a pivotal role in the pathogenesis of both COPD and IPF. However, it remains under debate how this common cellular process can be involved in these distinct lung diseases. At present, there are several potential scenarios for this conundrum. First, several aging-associated processes, including cellular senescence and telomere attrition [108], are common features of COPD and IPF; however, there are many distinct pathomechanisms among these diseases. For example, in addition to hallmarks of aging, IPF has several abnormal recapitulations of the developmental pathway, including Wnt signaling pathway [116,117,118] and Shh signaling [119]. Additionally, COPD and IPF have different patterns of microRNA dysregulations [120]. Second, COPD and IPF have distinct primary targets. While COPD generally targets small airway epithelial cells, which results in airway chronic inflammation [35], IPF targets AECs, which causes disruption of AEC-barrier integrity, insufficient for re-epithelialization and subsequent lung fibrosis [36,120,121,122]. In addition, mesenchymal precursor cell senescence, progressive decrease of matrix protein production, and subsequent emphysema might occur in COPD [123], as suggested by the enhanced senescence-related markers in mesenchymal cells of the lungs in patients with COPD [93]. Third, there is a distinctive genetic architecture and a divergent epigenetic dysregulation between the two diseases [120]. Fourth, different expression patterns of SASP factors exist as outputs of cellular senescence between COPD and IPF due to undetermined mechanisms, and that may lead to the distinct abnormal tissue remodeling [36,124]. As a whole, the reason why common age-related defective cell processes fall into distinct lung pathologies is currently unknown. Further understanding the pathomechanisms of these two diseases—including the machinery of repair process, epithelial–mesenchymal interaction, and the involvement of immunosenescence—may help in the establishment of new therapeutic strategies against devastating disorders.

## 5. Conclusions

**Figure 1:**
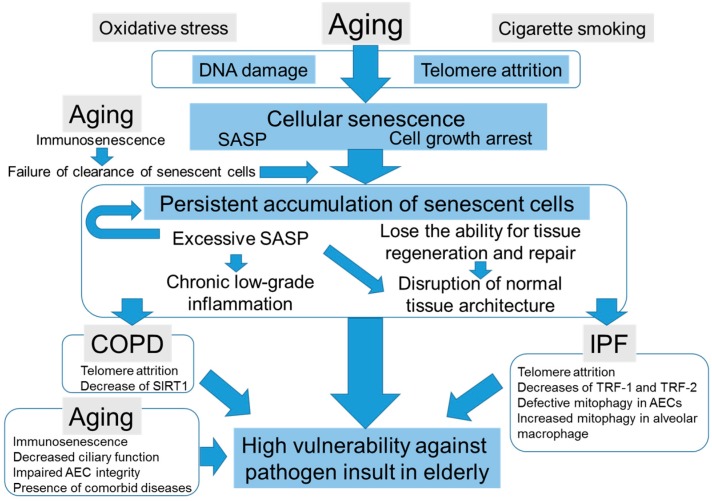
The correlation diagram among aging, cellular senescence, chronic obstructive pulmonary disease (COPD), idiopathic pulmonary fibrosis (IPF), and elderly pneumonia.

In this review, we discuss the impacts of cellular senescence in elderly pneumonia (Figure 1). Cellular senescence plays a pivotal role in tissue repair, tissue renewal, normal embryonic development, and antitumor effects. However, persistent cellular senescence and excessive SASP induce disruption of normal tissue microenvironments and chronic low-grade inflammation that result in increased susceptibility to infection in elderly. Thus, for elderly populations, antisenescent therapy may help eliminate senescent cells and subsequently improve resistance against pathogen insult. Antisenescent therapy might also prevent the development of senescent-related lung diseases, COPD and IPF. On the other hand, it is easy to assume that antisenescent therapy may induce considerable adverse effects, including cancer initiation and progression. Thus, it is important to investigate the precise mechanisms and roles of cellular senescence in the pathogenesis of different pathologies. The establishment of tissue-specific and cell-type-specific antisenescent therapy may also open new avenues for the development of attractive therapeutic strategies against these intractable diseases.

## Abbreviations

TNF	Tumor necrosis factor
IL	Interleukin
SASP	Senescence-associated secretory phenotype
ECM	Extracellular matrix
COPD	Chronic obstructive pulmonary disease
IPF	Idiopathic pulmonary fibrosis
SAβGAL	Senescence-associated β-galactosidase
TGF-β1	Transforming growth factor-β1
IGFBP	Insulin-like growth factor 1 binding protein
CDK	Cyclin-dependent kinase
NF-κB	Nuclear factor-κB
MMP	Matrix metalloproteinase
AEC	Alveolar epithelial cell
TLR	Toll like receptor
SIRT1	Sirtuin 1
TRF	Telomere repeat binding factor
PINK1	PTEN-induced putative kinase 1
